# An Atypical Case of Antineutrophil Cytoplasmic Antibody (ANCA)-Associated Vasculitis With Isolated Lower Airway Involvement in a Young Adult

**DOI:** 10.7759/cureus.117810

**Published:** 2026-10-12

**Authors:** Mitchell T Wong, Jiehuan Liu, Arianna Rodriguez, George Cheng, Paul Hsu, Alan Moazzam

**Affiliations:** 1 Department of Internal Medicine, Santa Clara Valley Health and Hospital System, San Jose, USA; 2 School of Biological Sciences, University of California San Diego, San Diego, USA; 3 Department of Pulmonary and Critical Care Medicine, UC San Diego Health System, San Diego, USA; 4 Department of Interventional Pulmonology, UC San Diego Health System, San Diego, USA; 5 Division of Rheumatology, Autoimmunity, and Inflammation, UC San Diego Health System, San Diego, USA; 6 Department of Hospital Medicine, UC San Diego Health System, San Diego, USA

**Keywords:** adolescent and young adult, airway fibrosis, anca-associated vasculitis, bronchial stenosis, bronchial webbing, bronchoscopic dilation, granulomatosis with polyangiitis, young adult

## Abstract

Antineutrophil cytoplasmic antibody (ANCA)-associated vasculitides (AAV) typically present in middle-aged adults with multiorgan involvement. We describe the case of a 19-year-old woman with asthma, progressive dyspnea, and recurrent bronchial stenosis and webbing. Serological testing showed rising perinuclear ANCA (p-ANCA) and anti-myeloperoxidase (MPO) antibody titers, whereas cytoplasmic ANCA (c-ANCA) and anti-proteinase 3 (PR3) antibodies were negative. Biopsy revealed nonspecific inflammation and fibrosis. Infectious causes, malignancy, relapsing polychondritis, and sarcoidosis were excluded. Although the presentation did not meet the 2022 American College of Rheumatology/European Alliance of Associations for Rheumatology (ACR/EULAR) classification criteria threshold for granulomatosis with polyangiitis (GPA), which are intended for research classification rather than clinical diagnosis, bronchial-limited AAV, most consistent with GPA, was considered the working diagnosis. The patient improved following corticosteroid treatment and bronchoscopic dilation.

## Introduction

Antineutrophil cytoplasmic antibody (ANCA)-associated vasculitides (AAV) are a group of rare, necrotizing small-vessel vasculitides comprising granulomatosis with polyangiitis (GPA), microscopic polyangiitis (MPA), and eosinophilic granulomatosis with polyangiitis (EGPA). These disorders typically present in middle-aged or older adults, with mean ages at diagnosis of 56.1 years for GPA and 67.7 years for MPA, and are characterized by multiorgan involvement, most commonly affecting the kidneys and respiratory tract [1]. GPA is distinguished by necrotizing granulomatous inflammation. Ear, nose, and throat (ENT) involvement occurs in up to 70% of patients with GPA and is frequently present at diagnosis. It has been associated with younger age at diagnosis, a relatively milder disease course, and improved survival [2,3]. Tracheobronchial involvement occurs in approximately 12%-23% of patients with GPA, with a reported mean age of 31 years at diagnosis [4,5]. It may lead to recurrent airway stenosis requiring repeated bronchoscopic interventions. Isolated bronchial involvement is particularly uncommon and can pose substantial diagnostic challenges. Three recently reported cases of pulmonary-limited GPA involving the lung parenchyma illustrate the diverse presentations of the disease: recurrent lung abscess [6], isolated lung nodules that subsequently progressed to multiorgan disease [7], and pulmonary parenchymal disease with hilar and mediastinal lymphadenopathy [8]. Bronchoscopic biopsy of endobronchial lesions establishes a definitive tissue diagnosis in only approximately 25% of GPA cases, according to general reviews of the literature. Consequently, diagnosis can be particularly challenging in the absence of multiorgan involvement, supportive serological findings, or diagnostic histopathology. Nevertheless, biopsy remains valuable for excluding alternative diagnoses [9].

We present the case of a 19-year-old woman with progressive, recurrent bronchial stenosis and serological evidence of AAV, without renal involvement or other systemic manifestations. This case highlights the diagnostic and therapeutic challenges of atypical, organ-limited AAV. To our knowledge, it is among the few reported cases of AAV presenting solely as bronchial webbing and stenosis in a patient younger than 20 years.

## Case presentation

Patient history

A 19-year-old woman with a history of mild intermittent asthma, well controlled with albuterol, presented with a two-month history of progressive exertional dyspnea and dry cough. She had never smoked cigarettes and reported a one-month history of vaping several years earlier. She denied other inhalational exposures and had no relevant family history or known drug allergies. She also denied fever, chills, hemoptysis, hematuria, joint or muscle pain, nasal deformities, skin rashes, and lower-extremity edema.

Eight months before the current admission, she had presented with similar symptoms and was found to have left mainstem bronchial stenosis and webbing on bronchoscopy. She underwent bronchial dilation and received corticosteroid therapy, which provided temporary symptomatic relief. The diagnostic workup for infectious, malignant, and autoimmune causes revealed a p-ANCA titer of 1:40 and an MPO antibody level of 5.1. Her current presentation represented a recurrence of symptoms following the initial treatment course.

Physical examination and laboratory evaluation

Physical examination revealed mildly diminished breath sounds bilaterally with wheezing. No skin rashes, joint swelling, nasal deformities, or lower-extremity edema were observed. Review of systems was unremarkable for ENT symptoms, including nasal crusting, epistaxis, hearing changes, and voice changes. Urinalysis showed bland sediment, and the serum creatinine level was 0.54 mg/dL, consistent with her established baseline.

The patient's dyspnea had worsened in the months preceding her initial admission. Serial laboratory evaluations over the nine-month period between her first and second admissions showed an increase in the erythrocyte sedimentation rate (ESR) from 2 to 47 mm/h (reference range: 2-37 mm/h). c-ANCA were undetectable, and the anti-PR3 antibody level was less than 1 U/mL (reference range: <2 U/mL). In contrast, the p-ANCA titer increased from 1:40 to 1:160 (reference range: <1:20), while the anti-MPO antibody level increased from 5.1 to 9.8 U/mL (positive threshold: >5 U/mL) over the same period. The absolute eosinophil count remained within normal limits. A comprehensive infectious workup, including testing for respiratory viral pathogens, tuberculosis, histoplasmosis, cryptococcosis, and coccidioidomycosis, was negative. The key laboratory findings are summarized in Table 1.

**Table 1:** Key laboratory findings ESR: erythrocyte sedimentation rate, c-ANCA: cytoplasmic antineutrophil cytoplasmic antibody, anti-PR3: anti-proteinase 3, p-ANCA: perinuclear antineutrophil cytoplasmic antibody, anti-MPO: anti-myeloperoxidase antibody.

Test	Result/value
Urinalysis/creatinine	Bland sediment/0.54 mg/dL (baseline)
ESR	2 → 47 mm/hr over nine months (reference: 2-37)
c-ANCA	Not detected
Anti-PR3 antibody	<1 U/mL (reference: <2)
p-ANCA	1:40 → 1:160 over nine months (reference: <1:20)
Anti-MPO antibody	5.1 → 9.8 U/mL over nine months (positive: >5)
Infectious workup	Negative (viral respiratory, tuberculosis, *Histoplasma*, *Cryptococcus*, *Coccidioides*)

Imaging

Computed tomography (CT) of the chest demonstrated progressive narrowing of the left mainstem bronchus to 1.5 mm, with additional mild stenosis of the right lower lobe segmental bronchi, which is not shown in Figure 1. No pulmonary nodules, ground-glass opacities, or interstitial lung disease were identified.

**Figure 1:**
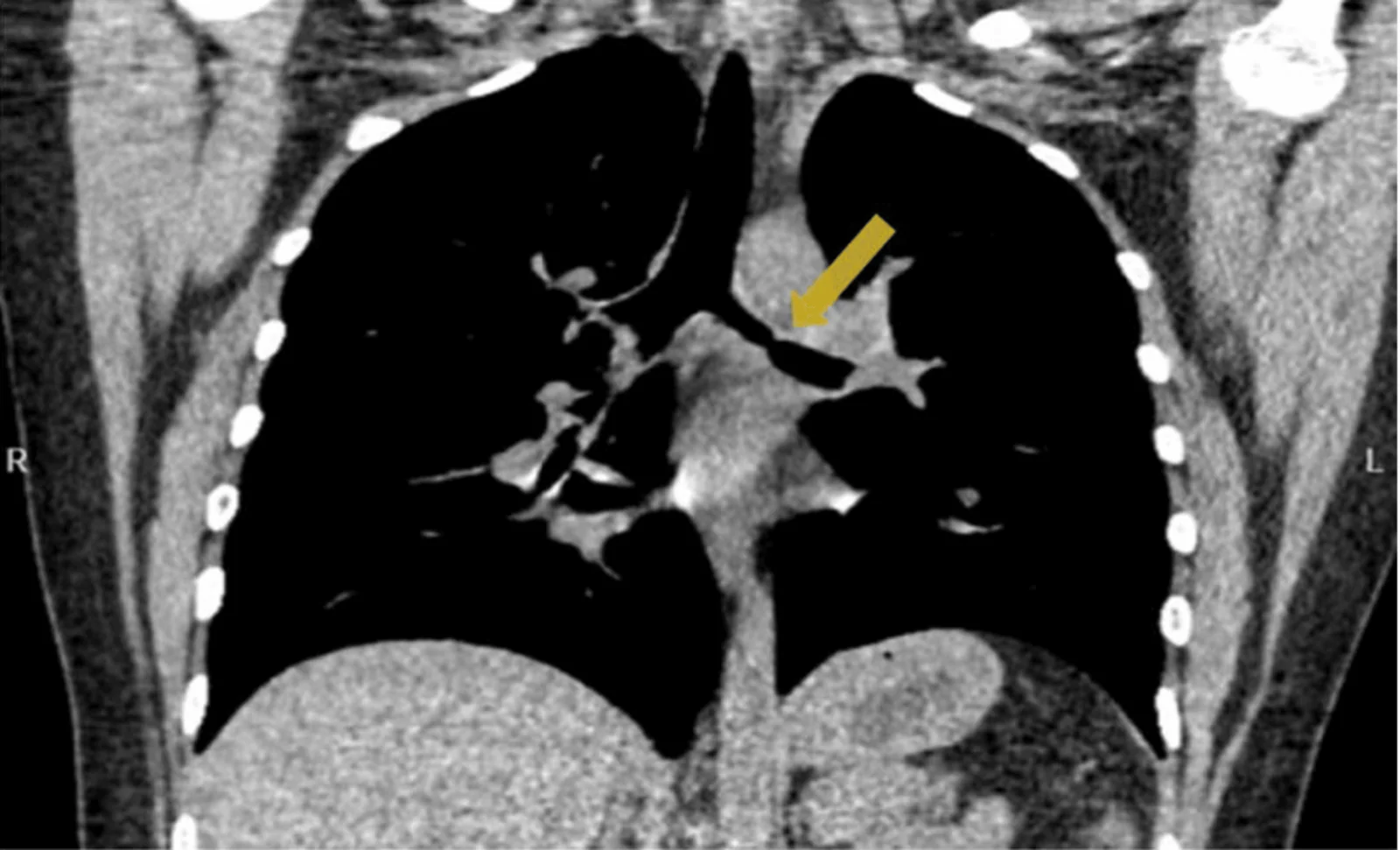
Coronal chest CT image demonstrating stenosis of the left mainstem bronchus (yellow arrow)

Bronchoscopy and biopsy

Flexible bronchoscopy revealed web-like mucosal structures and stenosis in the right anterior segmental bronchus (B3), as viewed from the right upper lobe bronchus (Figure 2A, 2B). Severe narrowing of the left mainstem bronchus was also observed. Bronchoscopic dilation was performed to relieve the stenotic segments and web-like lesions.

**Figure 2:**
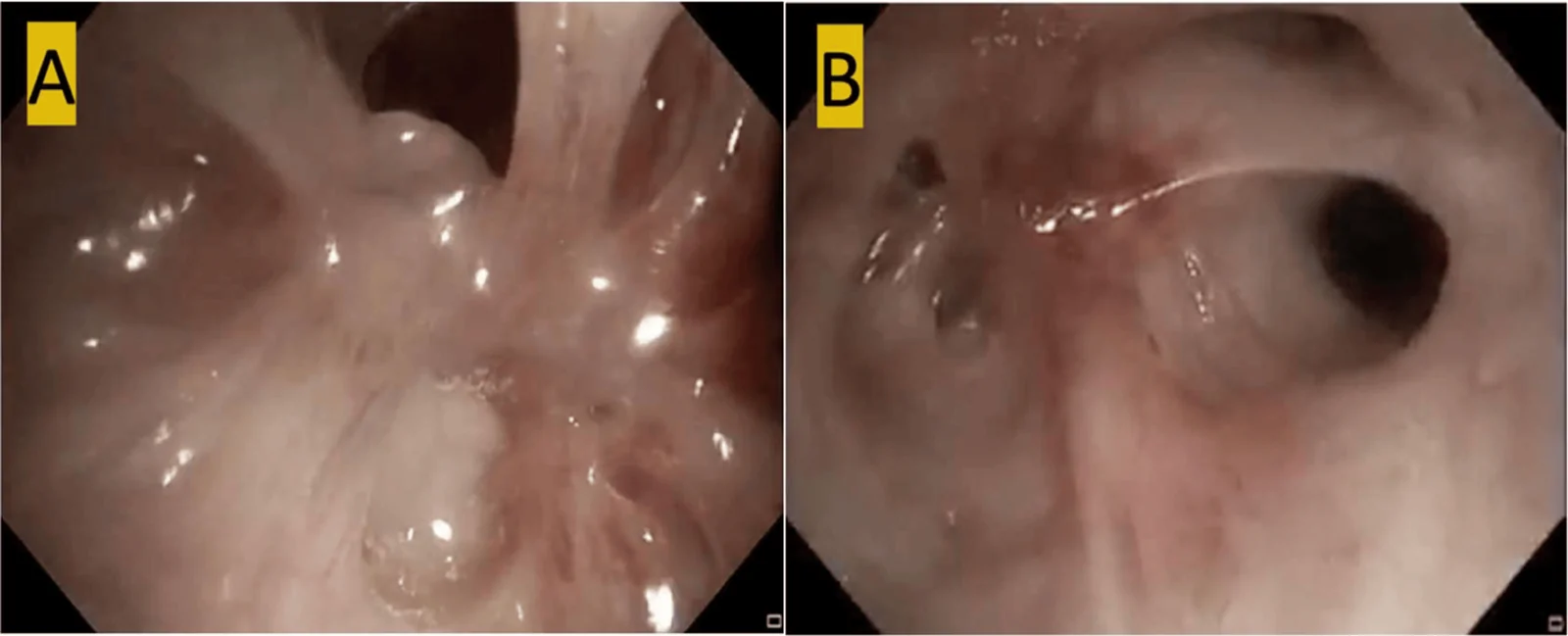
(A) Bronchoscopic view demonstrating web-like mucosal structures in the right anterior segmental bronchus (B3). (B) Bronchoscopic view from the right upper lobar bronchus demonstrating stenosis of the right anterior segmental bronchus (B3) prior to intervention

Bronchoalveolar lavage (BAL) cytology revealed no atypical or malignant cells. Biopsies of the bronchial walls at the stenotic segments and web-like lesions demonstrated acute and chronic inflammation with fibrosis. Gomori methenamine silver (GMS) staining was negative for fungal organisms. Follow-up bronchoscopy confirmed improved patency of the right anterior segmental bronchus (Figure 3).

**Figure 3:**
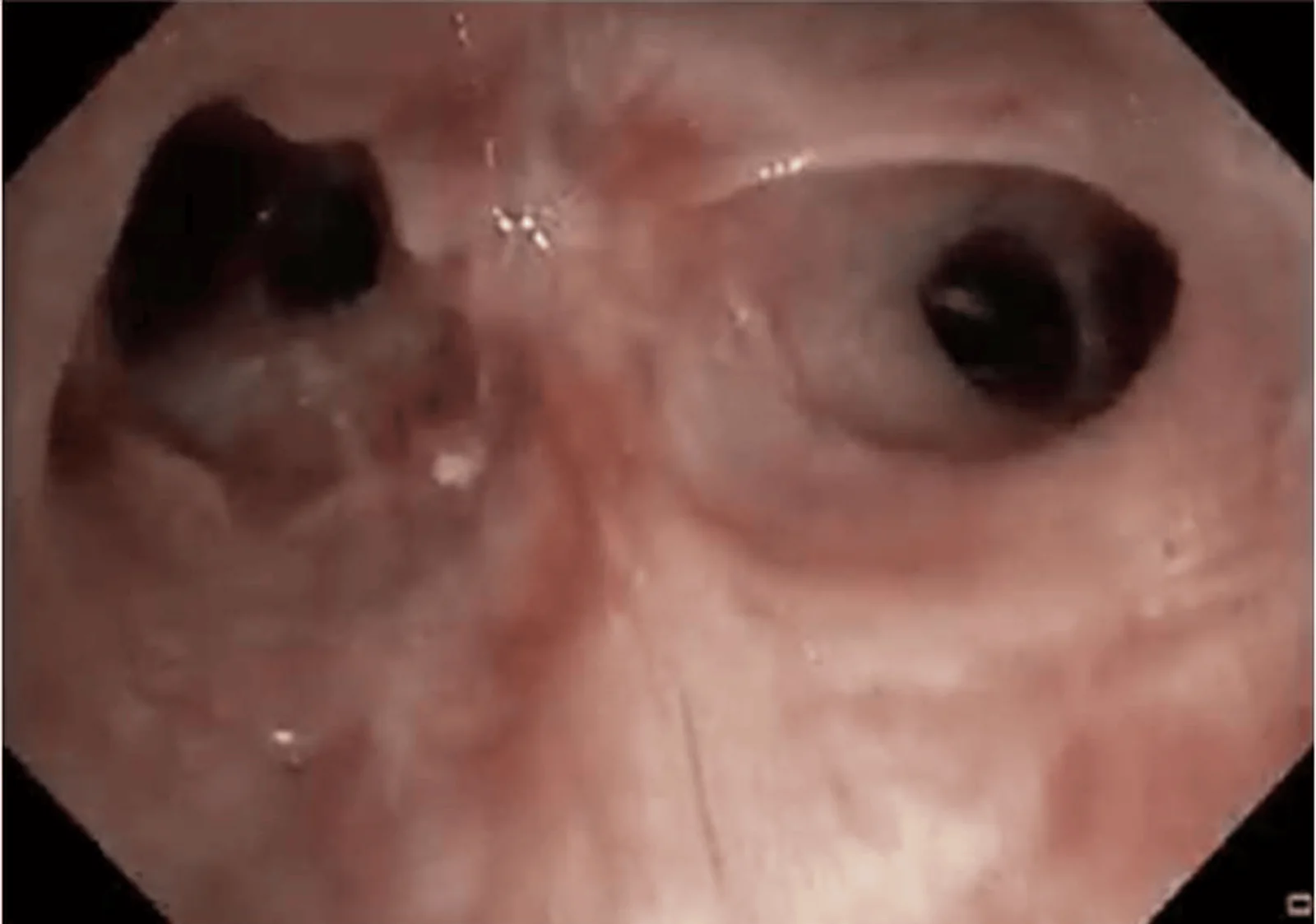
Bronchoscopic view from the right upper lobar bronchus demonstrating improved patency of the right anterior segmental bronchus (B3) following bronchoscopic dilation

Clinical course, diagnosis, and management

Based on the combination of recurrent bronchial stenosis and webbing, a normal absolute eosinophil count, rising p-ANCA and anti-MPO antibody titers, an elevated and rising ESR, negative infectious and malignancy workups, and nonspecific but compatible histopathological findings, a working diagnosis of bronchial-limited AAV, most consistent with GPA without renal involvement, was established. EGPA was considered less likely because of the normal absolute eosinophil count and the presence of recurrent bronchial stenosis and webbing.

The patient was treated with intravenous methylprednisolone at a dose of 1 g daily for three days, followed by oral prednisone at 1 mg/kg/day with a gradual taper. Rituximab was deferred because of the absence of definitive histopathological evidence of vasculitis and concerns regarding the risks of immunosuppression, including infection and potential reproductive health implications, given the patient's age. She was discharged in stable condition on an oral corticosteroid taper, with outpatient rheumatology follow-up arranged for further evaluation and management.

At the two-week follow-up, the patient reported resolution of dyspnea and an ability to exercise without limitation. Repeat bronchoscopy with cryospray therapy was scheduled for six weeks after discharge; however, she did not return for the planned procedure. During a brief telephone follow-up nine months later, she denied worsening dyspnea but reported a mild increase in cough. She expressed plans to follow up with her outpatient rheumatologist and pulmonologist.

## Discussion

GPA is a necrotizing granulomatous small-vessel vasculitis that classically affects the upper and lower respiratory tracts and kidneys. Severe pulmonary manifestations, including diffuse alveolar hemorrhage, are life-threatening emergencies that require urgent immunosuppressive treatment. According to the 2021 American College of Rheumatology (ACR)/Vasculitis Foundation guidelines, standard induction therapy for severe GPA generally includes high-dose glucocorticoids, such as intravenous methylprednisolone (500-1000 mg daily for three days), followed by oral prednisone at approximately 1 mg/kg/day (maximum 80 mg) with a gradual taper, in combination with rituximab or cyclophosphamide. Rituximab is conditionally preferred over cyclophosphamide for severe disease because of its efficacy and toxicity profile. Maintenance therapy commonly includes rituximab or azathioprine, with the duration individualized according to disease activity, relapse risk, and treatment tolerance. In this case, rituximab was deferred because of diagnostic uncertainty and the absence of clearly established severe, organ-threatening systemic vasculitis. Glucocorticoids and bronchodilators were used to manage the suspected inflammatory process and relieve airway obstruction.

The differential diagnosis included autoimmune, infectious, and malignant conditions. An autoimmune etiology was supported by the rising p-ANCA and anti-MPO antibody levels, progressive airway stenosis, and clinical improvement following high-dose glucocorticoid treatment during both admissions. Infection was considered less likely following a negative workup for viral respiratory pathogens, tuberculosis, and fungal infections. Malignancy was also considered less likely because BAL revealed no atypical or malignant cells, although this finding alone does not definitively exclude malignancy. Relapsing polychondritis was considered unlikely in the absence of characteristic auricular, nasal, or articular manifestations. Sarcoidosis was also considered but was not supported by the absence of characteristic thoracic imaging findings or granulomas on biopsy. EGPA was considered less likely given the normal absolute eosinophil count and the predominance of recurrent bronchial stenosis and webbing, although these findings do not independently exclude EGPA. MPA was another consideration because the p-ANCA/anti-MPO serological profile is commonly associated with MPA. However, the recurrent endobronchial stenosis and webbing were more suggestive of the recognized tracheobronchial manifestations of GPA, although this pattern is not specific to GPA.

Application of the 2022 ACR/EULAR GPA classification criteria yielded a score of -1, below the threshold of ≥5 required for classification. These criteria are intended for research classification after a diagnosis of small- or medium-vessel vasculitis has been established and should not be used in isolation to confirm or exclude a clinical diagnosis. In this case, p-ANCA/anti-MPO positivity contributes -1 point, while no additional scored features were identified: c-ANCA/anti-PR3 positivity, granulomatous inflammation, renal involvement, characteristic ENT manifestations, and pulmonary nodules were absent. Several findings therefore limit diagnostic certainty. Histopathology demonstrated nonspecific inflammation and fibrosis without granulomas, giant cells, or histological evidence of vasculitis. There was also no renal or upper respiratory tract involvement, and the anti-MPO-dominant serological profile was more suggestive of MPA than GPA. Accordingly, the working diagnosis of bronchial-limited AAV, with GPA as the leading consideration, was based on the compatible clinical and bronchoscopic findings, rising anti-MPO antibody levels, progressive airway stenosis on serial imaging, and exclusion of alternative etiologies. Such cases remain diagnostically challenging when serological and histopathological findings are insufficiently definitive, as illustrated by Teames et al. [10].

Several additional features warrant consideration. Isolated bronchial involvement as the sole manifestation of AAV is uncommon. Pulmonary-limited GPA more frequently involves the lung parenchyma, although tracheobronchial disease is a recognized manifestation and has been reported in a substantial proportion of patients [11]. The atypical serological profile, with negative c-ANCA/anti-PR3 results and rising p-ANCA/anti-MPO levels, further complicates disease classification. The nondiagnostic biopsy is consistent with the limited sensitivity reported for bronchoscopic sampling of endobronchial lesions in GPA [9]. However, a nondiagnostic biopsy neither confirms nor excludes AAV, and the diagnosis must be interpreted in the context of the overall clinical, serological, radiological, and endoscopic findings. Tracheobronchial involvement may recur independently of other systemic manifestations, and recurrent stenosis can necessitate repeated bronchoscopic interventions [5]. Limited airway disease may also precede the development of additional organ involvement, underscoring the importance of continued surveillance.

The patient was lost to clinical follow-up after missing the planned repeat bronchoscopy at six weeks. Although her dyspnea had resolved at the two-week follow-up and she reported no worsening dyspnea during a telephone contact nine months later, the absence of repeat bronchoscopy, interval serological testing, and documented rheumatology follow-up represents an important limitation of this report. Consequently, the long-term course of the airway disease, persistence of serological abnormalities, and possibility of subsequent systemic involvement remain unknown.

## Conclusions

This case illustrates a presumptive AAV-associated bronchial inflammatory disorder in a young adult, with GPA as the leading diagnostic consideration, although definitive classification remains challenging. The key clinical lesson is that bronchial-limited AAV should be considered in young patients presenting with recurrent, unexplained bronchial stenosis, even when serological findings are atypical, biopsy results are nondiagnostic, and formal classification criteria are not met. This case also highlights the need to individualize treatment in organ-limited disease, particularly when the diagnosis remains uncertain and life-threatening manifestations are absent. Although standard induction therapy for severe GPA generally combines high-dose glucocorticoids with rituximab or cyclophosphamide, the risks and benefits of additional immunosuppression should be assessed on a case-by-case basis. Longitudinal follow-up with pulmonology and rheumatology is essential, including surveillance for renal involvement through urinalysis and serum creatinine measurement, assessment for ENT and ophthalmological manifestations, serial ANCA testing as clinically indicated, and repeat bronchoscopy to evaluate airway patency and guide further intervention.
